# Genetic Polymorphisms Associated with Obesity in Southeast Asian Populations: A Systematic Review without Meta-Analysis

**DOI:** 10.21315/mjms-03-2025-201

**Published:** 2025-12-31

**Authors:** Ubashini Vijakumaran, Nor Azian Abdul Murad, Muhammad Irfan Abdul Jalal, Chin Siok Fong, A Rahman A Jamal, Noraidatulakma Abdullah

**Affiliations:** UKM Medical Molecular Biology Institute (UMBI), Cheras, Kuala Lumpur, Malaysia

**Keywords:** obesity, overweight, gene polymorphism, single nucleotide polymorphisms, Southeast Asia

## Abstract

Obesity is a growing global public health challenge, with genetic factors playing a crucial role in its development. This review synthesises findings from Southeast Asian studies to investigate the association between gene polymorphisms and obesity risk across various ethnic populations. A comprehensive search of three databases, PubMed, Scopus, and Web of Science, initially retrieved 2,021 articles, from which 25 studies were meticulously selected based on stringent inclusion and exclusion criteria. The quality of the studies was assessed through the Newcastle-Ottawa Scale (NOS), a risk bias tool. These studies encompass 8,312 participants and examined 33 single nucleotide polymorphisms (SNPs). *UCP* polymorphism demonstrated a significant association with overall adiposity (OR = 2.02, *P* = 0.01) in Malaysian women, while the rs659366 *UCP2* was linked to weight gain in an Indonesian cohort. *LEP* variants were not significantly associated with obesity in Malaysians, and *FTO* variants showed mixed results, with rs9939609 (OR = 3.72, *P* = 0.009) and rs1421085 (OR = 3.22, *P* < 0.001) variants being associated with obesity and higher body mass index (BMI) in Indonesians, but no significant findings in Malaysians. These results emphasise the genetic diversity within Southeast Asia and the challenges in replicating genetic associations across populations. To address these inconsistencies and improve our understanding of obesity in Southeast Asia, there is a pressing need for more extensive and diverse cohort studies, complemented by comprehensive genome-wide association studies (GWAS), to identify robust obesity biomarkers in Southeast Asia.

## Introduction

Obesity is a complex metabolic disorder that has become a primary global health concern, with its prevalence rising steadily across diverse populations. As of 2022, approximately one in eight people worldwide is affected by obesity. The World Health Organization (2024) (1) reports that 2.5 billion adults are overweight, and 890 million individuals are classified as obese. While environmental factors undoubtedly play a significant role in this increase, obesity primarily results from the interplay of genetic predispositions and environmental influences. Genetic variants among individuals can affect their susceptibility and response to obesogenic environments (2). Typically, obesity is categorised into two types. Monogenic obesity is a rare condition caused solely by the impairment of genes inherited in a Mendelian pattern (3). Polygenic obesity, the more common type, results from the interaction between a myriad of genetic polymorphisms, epigenetic regulation, and environmental factors (4). The central nervous system and neuronal pathways that modulate food intake and subsequent energy metabolism are major regulators of body fat and weight in monogenic and polygenic obesity (2). The involvement of multiple pathways in obesity (5, 6) presents significant challenges in understanding the complex mechanisms underlying the condition. One of the most studied and critical among these is the leptin-melanocortin pathway, which regulates energy metabolism through approximately 60 genes expressed in the hypothalamic region (7).

A key component of this pathway is the leptin (*LEP*) which plays a pivotal role in energy balance and appetite regulation. *LEP* is expressed in adipocytes and encodes the leptin hormone, which acts on the hypothalamus to convert proopiomelanocortin (*POMC*) into alpha-melanocyte stimulating hormone (α-MSH), which suppresses appetite and food intake (8). Individuals with leptin resistance have impaired signalling in the leptin-melanocortin pathway, leading to increased food intake and reduced energy expenditure (9). Additionally, children with impaired leptin-melanocortin signalling pathways also exhibit disruptive eating patterns from early childhood, leading to severe early-onset obesity (10). Leptin regulates intracellular pathways such as *PI3K/ AKT/mTOR*, *JAK2/STAT3*, and *ERK/MAPK* to control cell proliferation, differentiation, survival, migration, and invasion (11, 12). The *MAPK* pathway, including the extracellular signal-regulated kinase (*ERK*) 1/2, c-Jun N-terminal kinase (*JNK*), and *p38 MAPK*, also plays a crucial role in the pathogenesis of obesity by regulating adipogenesis, glucose homeostasis, and thermogenesis (13, 14). Notably, elevated expression of *p38 MAPK* in human adipose tissue has been linked to hypertrophic adipocytes and associated with elevated levels of insulin, glucose, and triglyceride (15).

While these signalling pathways provide important insights into the biological mechanisms of obesity, understanding the genetic underpinnings is equally critical. Advances in molecular and population-based approaches have facilitated the identification of genetic variations linked to obesity risk. Initially, gene polymorphism was explored through candidate gene studies, which later expanded into more comprehensive genome-wide association studies (GWAS) (16). The first GWAS in 2007 reported the presence of a genetic polymorphism in the first intron of rs9930506 *FTO* that is positively associated with body mass index (BMI), (*P =* 8.6 × 10^−7^), hip circumference (in cm) (*P =* 3.4 × 10^−8^), and weight (in kg) (*P =* 9.1 × 10^−7^) (17). Another GWAS comprising 38,759 participants revealed that adults with homozygous A allele at rs9939609 *FTO* weight 3 kg more than those who do not have it, with 1.67 increased odds of obesity (18). To date, GWAS studies have discovered more than 300 loci associated with obesity traits (19). However, few genes, such as *ADRB3* (20), *MCR4* (21), *BDNF* (22), *LEPR* (23), *FTO* (24), *PCSK1* (25), *CNR1* (26), and *PPARG* (27), have replicable associations with obesity (2). A large meta-analysis reported 463 signals associated with body fat in 694,649 European individuals (27). Similarly, studies in Asian populations have identified several key loci; for instance, a GWAS in Taiwan reported 13 significant SNPs, including *FTO* and *RALGAPA1*, in 6,546,460 individuals (28), while six SNPs were reported in the Han Chinese population (29).

In addition to these common variants identified through population-level studies, rare mutations have also been implicated in severe obesity cases. One such example is the *ADCY3*, which regulates *MC4R* trafficking. A homozygous frameshift mutation (c.3315del; p. Ile1106Serfs) in exon 21 of *ADCY3* was recently reported in severely obese Pakistani children (30). Besides, a different *ADCY3* variant, homozygous c.1102G>A (p. Asp368Asn), was identified through whole-exome sequencing in 10-year-old Turkish children (31), which has been associated with early-onset obesity, insulin resistance, and neurodevelopmental issues (31). Additional gene variants from the same pathway, such as semaphorin 3A-G (*SEMA3A-G*), plexinA1–4 (*PLXNA1–4*), and neuropilin 1–2 (*NRP1–2*), were identified in the adult obese population (32). In 2020, the FDA approved the first *MC4R* agonist, setmelanotide (RM-493), for the treatment of severe obesity (33, 34), marking a major milestone more than two decades after *MC4R* was first identified as a therapeutic target. This highlights the translational potential of genetic discoveries into clinical practice, as seen with the approval of setmelanotide for MC4R-related obesity. However, such pharmacogenomic advancements have largely been derived from studies conducted in Western populations, raising concerns about their relevance and applicability in other regions.

The Southeast Asian region comprises Brunei, Myanmar, Cambodia, Timor-Leste, Indonesia, Laos, Malaysia, Philippines, Singapore, Thailand, and Vietnam (35). It has experienced a significant rise in obesity prevalence, projected to double from 2010 to 2030, affecting up to 45 million individuals (36). Given the region’s rich ethnic diversity, unique dietary patterns, and distinct genetic backgrounds, it remains unclear whether genetic risk factors and treatment responses observed in Western populations are applicable to Southeast Asians. This raises an important question: do population-specific genetic markers exist that better explain obesity risk and potentially guide personalised treatment strategies in this region?

Building on this need for region-specific genetic data, a GWAS meta-analysis performed on Singaporeans, Malays, and Asian Indians confirmed the association between the *FTO* and obesity (37). The study successfully replicated 13 loci previously identified in European cohorts and reported three novel SNPs (rs2287019, rs2241423, and rs516175) linked to increased BMI in these Asian populations. Interestingly, 16 loci found in European GWAS were absent in Southeast Asians, which could be attributed to genetic heterogeneity. These findings underscore the importance of tailoring genetic research to local populations.

Further supporting the relevance of *FTO* in Asian contexts, a meta-analysis of 26 studies concluded a significant association between *FTO* SNPs and obesity risk in the Chinese population (OR = 1.30; 95% CI: 1.19, 1.42; *P* < 0.001) (38). Similarly, in Asian Indians, a minor A allele of the *FTO*, rs9939609, was associated with increased obesity risk (OR = 1.15; 95% CI: 1.08, 1.21, *P* = 2.14 × 10^−5^ (39). In contrast to findings in other Asian populations, no association was reported between 31 variants of *FTO* SNPs and obesity traits in Malaysians (40). Similarly, although a meta-analysis from 39 studies revealed a significant association between rs1137101 *LEPR* and obesity under the homozygous model (GG vs AA: OR = 1.39; 95% CI: 1.12, 1.73, *P* = 0.003 in Caucasians and Asians (23), this association was not observed in the multi-ethnic Malaysian populations (41, 42).

These inconsistencies across ethnic groups highlight the complexity of genetic predisposition to obesity, which may differ widely due to ancestry diversity, environmental interactions, and population sampling differences in Southeast Asia. Hence, this review aims to map the current landscape of genetic polymorphisms associated with obesity in the Southeast Asian population. By identifying established findings and highlighting knowledge gaps, we hope to support future research tailored to the region’s unique genetic and environmental context.

## Methods

### Search Strategy

**Figure 1:**
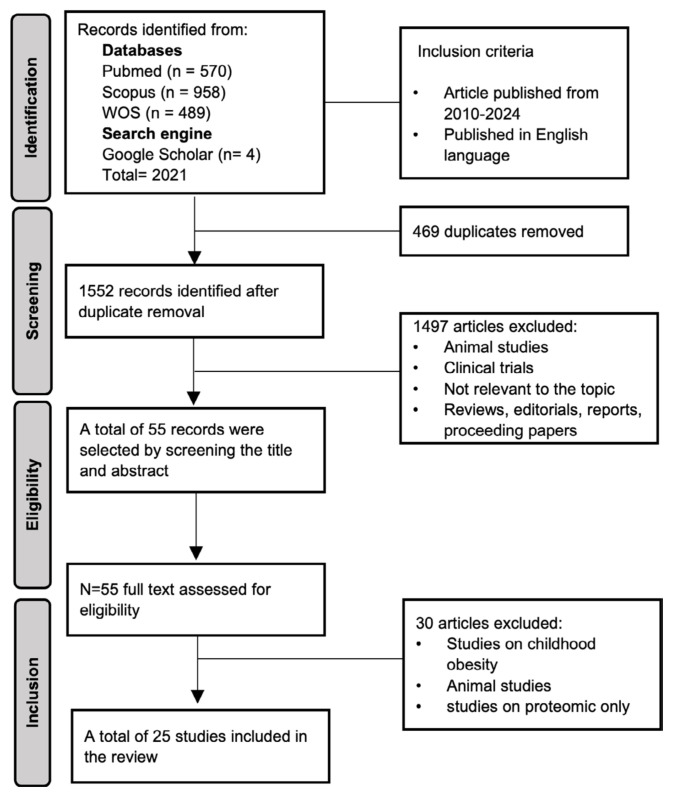
The PRISMA flow chart summarises the study retrieval, screening, and selection procedure

This systematic review followed the PRISMA (Preferred Reporting Items for Systematic Reviews and Meta-Analyses) 2020 and a systematic review without meta-analysis (SWiM) guidelines. The review has been registered under the International Prospective Register of Systematic Reviews (PROSPERO) (ID: CRD42024575327). The protocol can be assessed through https://www.crd.york.ac.uk/prospero/display_record.php?RecordID=575327. A combination of Medical Subject Headings (MeSH) terms was used for the search, including: 1) Adiposity; 2) Body Mass Index; 3) Obesity; 4) Overweight; 5) Gene Polymorphism; 6) Single Nucleotide Polymorphism; 7) SNPs; 8) Genome-Wide Association Studies; 9) GWAS; and 10) Southeast Asia. These terms were combined using Boolean operators (AND/OR) as follows: [(1 OR 2 OR 3 OR 4 OR 5 OR 6 OR 7 OR 8 OR 9) AND 10]. A total of 2,021 articles were retrieved from three electronic databases: Web of Science, PubMed, and Scopus. These articles were downloaded into EndNote 20.6 software, and 469 duplicate articles were removed. The titles and abstracts of the remaining studies were screened to select those relevant to gene polymorphism in the Southeast Asian population. The full texts of 55 studies were assessed for eligibility, and 25 were selected for review. The study retrieval and selection summary are presented in Figure 1 as a PRISMA flow chart.

### Selection Criteria

Full-text articles published in English within the past 15 years (2010–2024) were selected. The PECOS criteria for study inclusion and exclusion are detailed in Table 1. Only case-control, cross-sectional, and cohort studies were included; animal studies and clinical trials were excluded. The target population was Southeast Asians aged 18 to 70 years. Newborns, children, and individuals over 70 years old were excluded. Full texts of the studies were meticulously screened, and only those focusing on gene polymorphism related to obesity parameters were selected for data extraction.

### Data Extraction and Management

Data extraction was performed from 25 studies that passed the eligibility screening criteria. The following information was extracted and tabulated in Table 2. The following information was extracted:

- Gene, chromosome location, SNPs, and allele
- Country (population), study design, sample size, and sample characteristics (age and ethnicity)
- Genotyping method, definitions of obesity based on different BMI thresholds, and overall findings
- Odds ratio (95% CI), regression (*β*) coefficient (95% CI), and *P*-value

Significant heterogeneity was identified during data extraction, including the study design, type of SNPs, BMI thresholds for obesity, and population types. Hence, a SWiM was carried out per the published guideline (43). As per SWiM protocol, the populations, study design, and outcome were tabulated and illustrated via graphical presentation.

### Risk of Bias Grading Classification

The quality of the selected articles was assessed using the Newcastle-Ottawa Scale (NOS), a tool specifically designed for evaluating non-randomised studies. According to Wells et al. (66), the NOS has four main criteria for assessing the quality of studies screened for inclusion in meta-analyses: the selection of study groups, the comparability of study groups, the methods used and non-response rates of the participants for exposure assessment. The scoring system is as follows:

- High quality: Studies that score between 7 and 9 stars
- Fair quality: Studies that score between 4 and 6 stars
- Low quality: Studies that score between 1 and 3 stars

Two investigators were independently assigned to evaluate the quality of the identified studies based on the NOS scale.

## Results

### General Characteristics of the Included Studies

**Figure 2:**
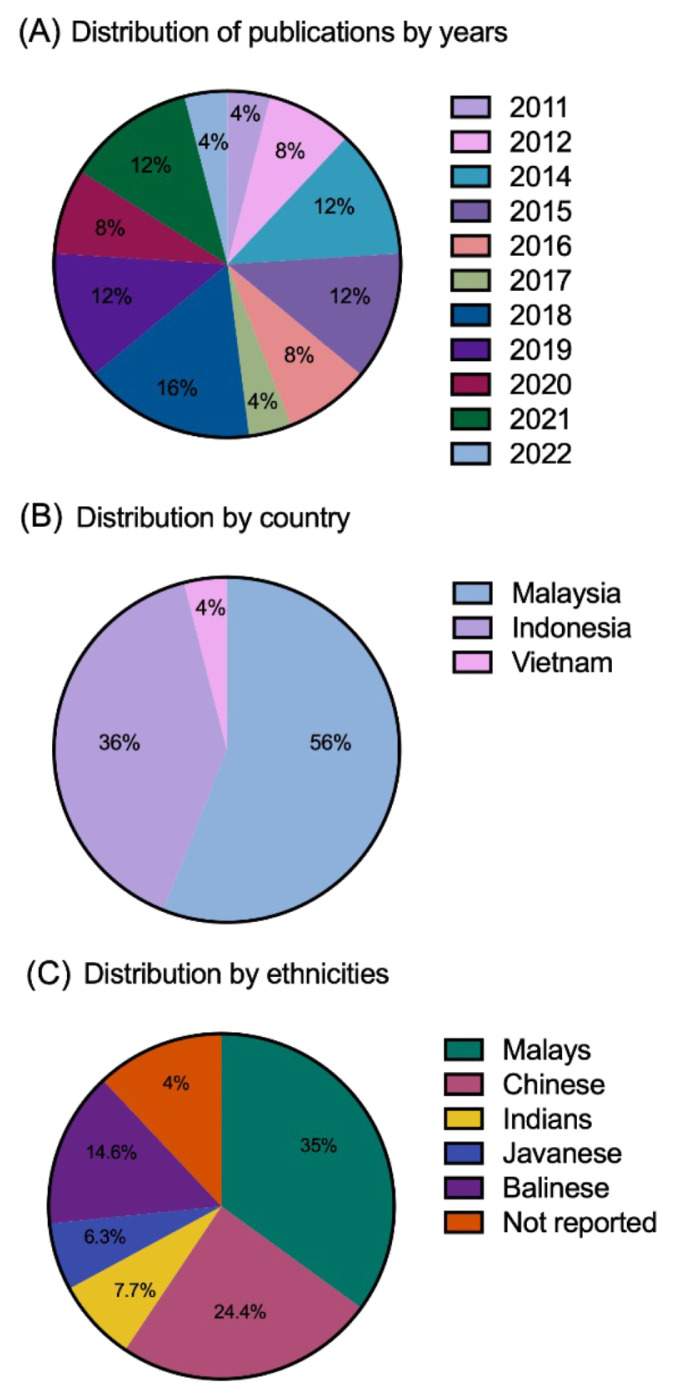
The distribution of the 25 selected studies by years, their respective populations, and ethnicities

**Figure 3:**
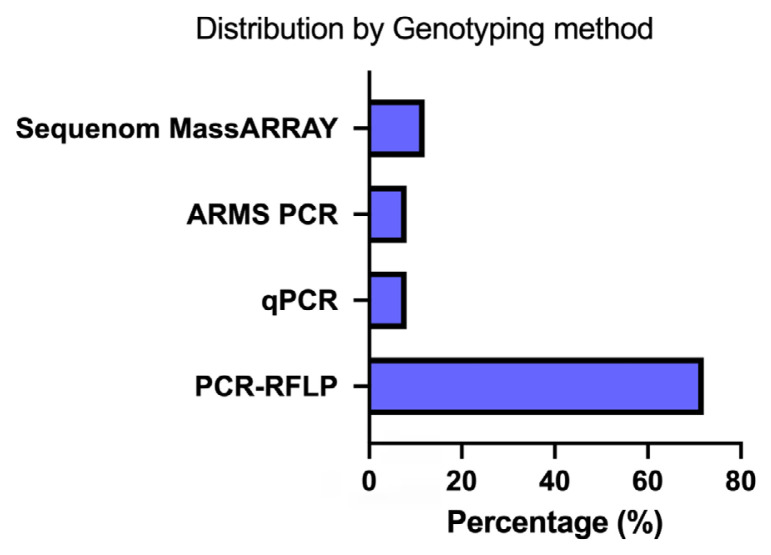
The distribution of genotyping methods and study designs among the 25 studies

Twenty-five studies published between 2010 and 2024 were selected through a systematic search. No studies were published in 2010, 2013, 2023, or 2024. The highest number of publications was in 2018, with four articles (16% of the total). Regarding the geographical distribution of research, 14 studies focused on Malaysians (40–42, 44–54), followed by 10 studies on Indonesians (55–64) and one study on Vietnamese (65). The combined population across the 25 studies consisted of 8,312 participants representing five ethnic groups: Malay, Chinese, Indian, Javanese, and Balinese. Malay participants comprised the largest group, comprising 35% of the total, with 2,909 individuals. This was followed by Chinese participants, who comprised approximately 24.4% of the total with 2,035 individuals, and Balinese participants, who represented 14.6% with 1,215 individuals. Notably, about 12% of participants across seven studies did not have their ethnicity disclosed, with these studies primarily conducted in Indonesia and Vietnam. A summary of the distribution of studies by year, country, and ethnicity is illustrated in Figure 2. This review included 25 studies, comprising 12 case-control studies, 12 cross-sectional studies, and one cohort study. Regarding genotyping methods, Polymerase Chain Reaction-Restriction Fragment Length Polymorphism (PCR-RFLP) was the most used, featured in 18 studies, accounting for 72% of the total. Amplification refractory mutation system (ARMS) PCR was used in three studies, quantitative PCR (qPCR) was used in two studies, and Sequenom MassArray^®^ was used in two studies. Figure 3 illustrates the summary of study designs and genotyping methods.

### Risk of Bias Evaluation

We evaluated the quality of the selected studies using the NOS. Of the 25 studies assessed, 18 were categorised as high quality, six as fair quality, and one as low quality. Sample size calculations were reported in the minority of studies (*n* = 9; 36%). One study (64) was rated as low quality, receiving only two stars due to the absence of data on the frequency of obesity among the obese and non-obese groups. The authors accumulated medical history through questionnaires from the participants, which showed ascertainment of exposure. The participants were postmenopausal women, who were not an accurate representation of the obese Vietnamese population. Additionally, six studies (44, 46, 50, 52, 53, 63) recruited participants from university students and staff, limiting the generalisability of the results to the broader population. Students’ unique lifestyles and environments do not reflect those of the general working adult population. Furthermore, due to the pervasive use of the convenience sampling method, several studies oversampled Chinese participants (44, 46, 50, 52, 53), which does not reflect the actual population’s demographic composition. Similarly, other studies focused exclusively on the Malay ethnicity (40, 45, 47, 48, 51), limiting the generalizability of their findings to the overall Malaysian population. Four studies (49, 55, 56, 65) relied on self-reported medical histories, which may introduce recall bias. One study (62) utilised BMI values on a continuous scale as the outcome measure, which is insufficient for predicting the associations between the investigated SNPs and the obesity category. Meanwhile, another study (51) did not report the anthropometric measurements of the participants, further limiting the study’s conclusions. The summary of the risk of bias assessment is presented in Tables 3, 4 and 5.

Due to the profound heterogeneity in the inter-study methodological quality, we did not proceed with meta-analysing the study results. Therefore, we opted for a narrative systematic review to synthesise the results from different studies.

## Discussion

The current review highlights several Southeast Asian studies investigating the association between obesity-related gene polymorphisms and obesity risk. This region offers a unique opportunity to understand how genetic variation, interacting with diverse environmental exposures and ethnic backgrounds, contributes to the complexity of obesity. Among the genes most extensively studied in the selected literature are *LEP* and *LEPR* (41, 42, 51, 60). Leptin, encoded by the *LEP*, plays a key role in regulating appetite, body weight, and fat metabolism (67). It is secreted primarily by white adipose tissue and acts by binding to its receptor, *LEPR*, in hypothalamic neurons, where it suppresses food intake and promotes energy expenditure (68, 69).

Several studies in Malaysia and Indonesia have evaluated the common *LEP* and *LEPR* variants, though findings have been inconsistent. For example, in a study of 408 Malaysians, Fan and Say (41) reported no significant association between *LEP* SNPs rs2167270 and rs7799039, as well as *LEPR* SNPs rs1137100 and rs1137101 with obesity risk. However, these variants were significantly associated with obesity in the Chinese populations. The lack of significant findings in the Indian subgroup was likely influenced by a smaller sample size, where the statistical power ranged from 57% to 63% (41). Similarly, Wan Rohani et al. (51) found no significant association between the *LEP* variants rs2167270 and rs7799039 and obesity in a study involving 249 Malays in Malaysia. Nevertheless, the same study reported that the AAG haplotype formed by three *LEP* SNPs, rs7799039, rs2167270, and rs4731426, was significantly associated with obesity, with an odds ratio (OR) of 8.89 (95% CI: 1.59, 49.78) (51). In contrast, another study involving 185 multi-ethnic Malaysians found no association between BMI and *LEP* rs7799039 (*P* = 0.117) and *LEPR* rs1137101 (*P* = 0.469), nor between leptin levels and these SNPs (*LEP* rs7799039: *P* = 0.196; *LEPR* Q223A: *P* = 0.453) (42). Conversely, a cross-sectional study from Indonesia found a positive association between rs1137100 and rs1137101 *LEPR* with both obesity and leptin levels (60). Specifically, obese individuals with the RR genotype of rs1137100 in the *LEPR* had leptin levels that were 99% higher, with an average BMI of 36.10, compared to controls with a BMI of 22.04. Additionally, obese individuals with the KK+KR genotype had leptin levels that were 28.3% higher, with an average BMI of 33.86, compared to controls with a BMI of 22.17 (60).

Unlike these positive associations observed in Indonesia and other populations, most studies in Malaysia have reported no significant links between *LEP* variants and obesity. This contradicts the findings from Finnish (70), Caucasians (71, 72), Saudi Arabian (73), and European populations (74), where *LEP* polymorphisms were associated with obesity traits. These inconsistencies may be attributed to small sample sizes, varying study designs, and the use of non-random purposive sampling, which limits the generalizability of findings (41, 51). Additionally, genetic factors such as differences in linkage disequilibrium patterns, allele frequencies, and population structure across ethnic groups may contribute to the divergent associations observed in obesity-related SNPs (75, 76).

Supporting this complexity, a meta-analysis of nine studies involving 2,988 participants concluded that the rs7799039 *LEP* polymorphism is not generally associated with the development of obesity. However, a significant association was observed in specific populations, including a small South American (*n* = 788) (77) and in the Tunisian (*n* = 329) population (78), where rs7799039 was associated with increased obesity risk, suggesting the influence of population-specific genetic factors. Similarly, *LEPR* polymorphisms have shown ethnic variation. In Caucasian populations, rs1137100 showed a significant association in both BMI and fat mass (*P* = 0.02 and *P* = 0.05), while rs1137101 was also significantly linked to BMI (*P* = 0.005 and *P* = 0.03) (79).

In addition, *FTO* variants, first identified as being positively associated with obesity in European populations (80), have been studied across four Asian ethnicities: Balinese Indonesians, Malays, Chinese, and Indians in Malaysia (40, 49, 55, 56, 59). A study conducted in Jakarta, although not specifying participants’ ethnicity, found that those with the AT/AA genotypes of rs9939609 *FTO* had a 3.72-fold higher risk of obesity (55). Similarly, the minor AA genotype of rs9939609 *FTO* increased BMI by 1.25 kg/m^2^ (*P* = 0.012), while the CC genotype of rs1421085 increased by 1.12 kg/ m^2^ (*P* = 0.022), particularly in females (59). The minor allele frequency (MAF) of this study was 0.19, consistent with findings from studies conducted among multi-ethnic Malaysians (81) and Minangkabau Indonesians (82). The study involving 275 Minangkabau Indonesians reported that the AA genotype of rs9939609 *FTO* has significantly higher body fat, weight, waist-hip ratio, and BMI of 27.39 (SD 4.69) compared to TT, which has a BMI of 23.96 (SD 4.51) and TA of about 24.56 (SD 4.8) (82). Additionally, the GG genotype of another rs9930506 *FTO* was found to be associated with obesity in the Malaysian population under a codominant model (49). The CC genotype of the rs1421085 variant in Indonesians was linked to a higher BMI of 12.58 kg/m^2^ (*P* = 0.001) in codominant and 12.38 kg/m^2^ (*P* < 0.001) in the recessive model compared with those without this variant, indicating a recessive trait (56). The MAF was 22%, lower than that observed in the Balinese population (59). However, a study involving 587 Malays failed to find an association between 31 *FTO* SNPs genotypes and allelic frequencies with obesity, except for rs17817288, which was significantly associated with LDL-C (40). Moreover, a GWAS study conducted among Singaporean Chinese also found no association between *FTO* variants and BMI (83). This highlights the possibility that ethnic-specific gene-environment interactions or other modifying loci may modulate the effect of *FTO* variants.

*UCP*, primarily located in mitochondria, plays a key role in regulating thermogenesis and energy expenditure while also offering protection against oxidative stress (84). The A/G genotype of rs1800592 *UCP* was the initial SNP established by Canadian scientists in 1994 to be associated with high BMI in individuals with a family history of obesity. In Southeast Asia, *UCP* polymorphisms were studied in three different populations, including Malaysia, Indonesia, and Vietnam (52, 53, 57, 63, 65). The combination of the rs1800592 *UCP1* AA genotype and the rs1800849 *UCP3* CC genotype has been notably associated with a higher waist-hip ratio (WHR) of 0.85 (SD 0.10) and a BMI of 26.17 (SD 5.27) compared to other genotype combinations such as AA/TT, GG/CC, and GG/TT, which are associated with lower BMIs of 22.11 (SD 3.01), 22.05 (SD 3.77), and 22.38 (SD 4.47), respectively, in the Malaysian Chinese population (52). Interestingly, the presence of the T allele of *UCP3* in the Chinese population is linked to a reduced risk of obesity, as carriers of the T allele exhibit a 30% lower risk of central obesity and a 2.5% lower WHR compared to those with the C allele (52). This suggests a potential protective effect of the T allele against obesity. Moreover, while *UCP1* alone is not significantly associated with WHR, its combination with *UCP3* appears to have a synergistic effect, significantly influencing obesity and adiposity. Meanwhile, unlike Malaysians, the G allele of rs1800592 *UCP1* was susceptible to weight gain compared to the A allele in the Finnish population (85). A similarly significant association was found for the minor G allele with BMI (OR: 1.52, CI: 1.10, 2.08, *P* = 0.009) in the Saudi Arabian population (86) and associated to fat mass (*P* = 0.002) and muscle mass (*P* = 0.019) in Mexican adults (87).

Furthermore, Say et al. (53) reported a significant association between the *UCP2* gene polymorphism and adiposity in Malaysian women. In the Chinese population, the MAF of *UCP2* was 0.12, consistent with findings in other Chinese cohorts (88). Among Indians, the MAF was higher, at 0.21, similar to data from southern Indians of Tamil Nadu, India (89). This difference may be attributed to the historical migration patterns and ancestral connections between Malaysian Indians and Chinese and their mainland counterparts. In contrast, the TT genotype and T allele of *UCP2* carry less risk of obesity compared to the C allele, as the OR CI of TT was 0.4 (95% CI: 0.13, 1.20), while the T allele was 0.55 (95% CI: 0.32, 0.95) in male Indonesian Javanese (63). Besides, an Indonesian Nutrigenetic Cohort reported that individuals with the GG genotype for rs659366 *UCP2* are associated with high energy intake, body fat, and weight (57), and the AA genotype has been positively associated with higher BMI in the rural Balinese population (64). Besides, a pairwise gene-gene interaction analysis of vitamin D receptor (*VDR* ApaI) and *UCP2* demonstrated a significant interaction (*P* = 0.003) and 69% predictive accuracy for overweight and obesity in postmenopausal Vietnamese women (65).

The *ADR2A* was first noted to have a crucial role in regulating insulin secretion and lipolysis (OR = 1.62; 95% CI: 1.06, 2.49; *P* = 0.026) in Swedish obese individuals without T2DM (90), with a stronger association with obesity in Swedish women with T2DM (OR = 7.61; 95% CI: 1.70, 34.17; *P* = 0.008) (91). In Malaysians, a gene-gene interaction between rs553668 *ADR2A* and the angiotensin-converting enzyme rs4646994 *ACE* polymorphisms was associated with lower central adiposity (44, 53). However, in a separate study in the Malay population, no significant association was found between the *ACE* variant and obesity parameters (48).

Based on the findings from these studies, several limitations and challenges were found that prevent the definitive ascertainment of SNPs associated with obesity and the generalisability of the results to the wider Southeast Asian population. First, there is a significant gap in replicating the results consistently across different populations and ethnic groups due to population-specific genetic factors. Second, the small sample sizes employed in many studies have also limited the study power to detect significant associations between SNPs of smaller effect sizes (e.g. smaller ORs) and obesity. Thus, inter-country collaboration within the Southeast Asian region may be leveraged to increase the number of study participants and improve study power. Besides, Southeast Asia has experienced rapid urbanisation and economic growth in recent decades, which has led to substantial changes in diet and physical activity patterns (36). These environmental shifts could exacerbate the impacts of genetic factors on obesity through gene-environment interaction, particularly among individuals carrying high-risk alleles (92). Furthermore, as reflected in Table 2, only three countries, Malaysia, Indonesia, and Vietnam, have conducted genetic association studies on obesity, highlighting a significant geographical research gap within the Southeast Asian region. This limited representation restricts the ability to generalise findings across the diverse ethnic and genetic backgrounds present in Southeast Asia.

## Conclusion

In conclusion, the reviews highlight the complex and multifaceted nature of obesity, particularly in the context of gene polymorphisms within Southeast Asian populations. The *LEP* and *UCP* polymorphisms are among the most studied in Southeast Asian populations. The *UCP* I/D polymorphism has been significantly associated with overall adiposity in Malaysian women, while in the Indonesian cohort, the GG genotype of *UCP2*, particularly the rs659366 variant, was linked to weight gain. On the other hand, *LEP* variants did not show a significant association with obesity in Malaysians. Similarly, the *FTO* shows population-specific effects. The rs9939609 variant was associated with obesity in Indonesians in two studies, and the rs1421085 variant was linked to a higher BMI. However, *FTO* variants were not significant determinants of obesity in the Malaysian population. These findings emphasise the potential of genetic polymorphisms to serve as population-specific biomarkers for obesity, underlining the importance of precision-based approaches in prevention and management.

Moreover, the findings underscore the importance of considering the genetic diversity and environmental factors contributing to obesity and the challenges in replicating genetic associations across different ethnic groups. Future research should prioritise large-scale, multi-ethnic genomic studies to identify robust and reproducible biomarkers. Such efforts can inform targeted public health strategies and early interventions. Cross-border collaboration, particularly among developing nations, could enhance the exploration of population-specific genetic factors related to obesity. Ultimately, these initiatives could facilitate the development of biomarker-driven risk assessments, screening tools, and personalised interventions tailored to the unique genetic and environmental context of Southeast Asia.

## Figures and Tables

**Table 1:** PECOS criteria of the studies

Criteria	Inclusion	Exclusion
Population	Southeast Asia participants 19 to 69 years old All genders and ethnicities	*In vitro* cell model Animals < 18 years old > 70 years old
Exposure	Combination of genetic susceptibility (e.g. candidate genes, genome-wide association studies, polygenic risk scores, genetic risk scores, single nucleotide polymorphisms) and obesity risk parameters	Studies that did not include both exposures
Comparison	Non-obese participants	-
Outcome	Obesity risk factor (weight-related anthropometric measurements [BMI, weight, waist circumference, waist-to-hip ratio, body fat])	Studies that failed to report the desired outcome
Study design	Cohort, case-control, cross-sectional	Clinical trials, including randomised and non-randomised, animal studies

**Table 2:** Summary of study characteristics and main findings of the selected articles

Gene and chromosome location	SNPs and alleles	Country (reference) Study design and sample size	Genotyping method Obese definition	Sample characteristics Age (years) Ethnicity	Findings	Highest OR 95% CI *P*-value
G: FTO L: 16q12.2	rs1077128 rs11643744 rs7186521 rs13334933 rs16952517 rs6499643 rs4784323 rs7206790 rs9939973 rs1421085 rs1558902 rs10852521 rs16952522 rs17817288 rs1121980 rs16945088 rs17817449 rs8050136 rs9935401 rs3751812 rs9939609 rs7190492 rs7204609 rs17218700 rs11642841 rs1861867 rs11075994 rs1421090 rs17818902 rs7191513	Malaysia (40) Cross-sectional *N* = 587 Obese *n* = 158 Non-obese *n* = 429	Sequenom MassARRAY^®^ iPLEX Platform (MALDI-TOF) ≥ 30.0 kg m^2^	48.29 ± 9.89 Malay	rs17817288 was significantly associated with LDL-C No significant difference in genotype or allelic frequency for SNPs between obese and normal groups	NR
G: *LEP* A19G L: 7q32.1	rs2167270	Malaysia (41) Cross-sectional *N* = 408 Obese *n* = 190 Non-obese *n* = 218	PCR-RFLP > 27 kg/m^2^	52.4 ± 13.7 Malay = 148 Chinese = 177 Indian = 83	Not all SNPs are associated with obesity among Malaysians	OR = NR
G: *LEP* G2548A L: 7q32.1	rs7799039					
G: *LEPR* K109R L: 1p31.3	rs1137100					
G: *LEPR* Q223R L:1p31.3	rs1137101					
G: *LEP* G2548A L: 7q32.1	rs7799039	Malaysia (42) Cross-sectional *N* = 185 Obese *n* = 95 Non-obese *n* = 87	PCR-TaqMan > 29.9 kg/m^2^	20.84 ± 2.2 Malay = 61 Chinese = 45 Indian = 56 Others = 23	No significant association was found between ethnic groups with *LEP*, G2548A and *LEPR*, Q223A *LEPR* polymorphism showed no association with plasma leptin Overweight and obese participants were in stage I of leptin resistance	LEPR, Q223R of AA genotype association with obesity OR = 2.50 CI = 0.11, 59.9 *P* = 0.50
G: *LEPR* Q223R L: 1p31.3	rs1137101					
G: *ADRA2A* L: 10q25.2	rs553668	Malaysia (44) Cross-sectional *N* = 214 Obese *n* = 142 Non-obese *n* = 72	PCR-RFLP ≥ 25 kg/m^2^	26.27 ± 11.93 Malay = 45 Chinese = 116 Indian = 53	People with both ADRA2A, rs553668 GG and ACE I/D II significantly has lowest WHR, could be due to gene-gene interaction	*ACE* associated with central adiposity OR = 2.02 CI = 0.87, 4.70 *P* = 0.10
G: *ACE* L: 17q23.3	rs4646994					
G: *ADRB2* L: 5q32	rs1042714	Malaysia (45) Cross-sectional *N* = 672 Obese *n* = 17 Non-obese *n* = 500	PCR-Taqman SNP > 90 cm for men > 80 cm for women	48.22 ± 10.05 Malay	rs1042714 was significantly associated with log-transformed HDL-C, DBP, and TG No association found towards BMI and obesity	OR = NR
G: *DRD2* Taq1A L: 11q23.2	rs1800497	Malaysia (46) Case-control *N* = 394 Obese *n* = 67 Non-obese *n* = 327	PCR-RFLP ≥ 25 kg/m^2^	52.4 ± 13.7 Chinese = 308 Indian = 86	*DRD2*, Taq1 SNPs were not associated with adiposity and obesity, but have influenced eating behaviour	*DRD2*, Taq1 A genotype association with WHR OR = 1.19 CI = 0.57, 2.47 *P* = 0.64
G: *DRD2* L: 11q23.2	rs1079597					
G: *DRD2* Taq1D L: 11q23.2	rs1800498					
G: *ADIPOQ* L: 3q27.3	rs17366568	Malaysia (47) Cross sectional *N* = 574 Obese *n* = 150 Non-obese *n* = 424	Sequenom MassARRAY TaqMan PCR ≥ 30 kg/m^2^	45.89 ± 6.34 Malay	*ADIPOQ* rs17366568 significantly associated with obesity, while rs3774261 not significantly associated Genotypes AG and AA of rs17366568 significantly higher in obese group	A allele in obese group OR = 2.15 CI = 1.13, 4.09 *P* = 0.024
	rs3774261					
G: *ACE* L: 17q23.3	Insertion/ deletion (I/D)	Malaysia (48) Cross sectional *N* = 219 Obese *n* = 94 Non-obese *n* = 123	PCR-RFLP ≥ 30 kg/m^2^	35.22 ± 10.10 Malay	*ACE* gene I/D polymorphism is not associated with obesity and obesity-related phenotypes	ACE ID genotype association with obesity OR = 1.32 CI = 0.75, 2.34 *P* = 0.335
G: *FTO* L: 16q12.2	Rs9930506	Malaysia (49) Cross-sectional *N* =178 Obese *n* = 79 Non-obese *n* = 99	PCR-RFLP ≥ 27.5 kg m^2^	41.7 ± 0.9 Chinese = 42 Malay = 86 Indian = 50	rs9930506 (G) not associated with obesity in dominant model but associated with codominant model	rs9930506, GG, associated with obesity in codominant model OR = 2.87 CI = 1.14, 7.19 *P* = 0.02
G: *IL1RA* intron 2 L: 2q14.1	NR	Malaysia (50) Case-control *N* = 315 Obese *n* = 54 Non-obese *n* = 261	PCR-RFLP ≥ 25 kg/m^2^	20.6 Malay = 23 Chinese = 251 Indian = 41	Individuals with *IL1RA* I/II genotype or allele II showed higher risk of having adiposity	IL1RA, I/II OR = 12.21 CI = 2.54, 58.79 *P* = 0.002
G: *IL4* L: 5q31.1	NR					
G: *LEP* G2548A L: 7q32.1	rs7799039	Malaysia (51) Case-control *N* = 249 Obese *n* = 148 Non-obese *n* = 101	PCR-RFLP ≥ 25 kg/m^2^	Age: NR Malay	No significant single association between SNPs and BMI and obesity Haplotype AAG of G2548A, H1328080, and A19G reported to have significant association with obesity	AAG haplotype association with obesity OR = 8.89 CI = 1.59, 49.78 *P* = 0.002
G: *LEP* A19G L: 7q32.1	rs2167270					
G: Leptin H1328080 L: chr7 128238282	rs12535747					
G: *UCP1* L: 4q31.1	rs1800592 (A/G)	Malaysia (52) Case-control *N* = 447 Obese *n* = 111 Non-obese *n* = 336	PCR-RFLP ≥ 25 kg/m^2^	24.66 ± 6.90 Malay = 46 Chinese = 339 Indian = 62	Lack of association between single UCP1/3 and obesity Combination of UCP1 A allele and UCP3 C allele associated with WHR Combination of *UCP1* AA and *UCP3* CC genotypes showed elevated BMI	T allele had significantly less risk of obesity in Chinese OR = 5.69 CI = 5.48, 1.00 *P* = 5.04
G: *UCP3* L: 11q13.4	rs1800849 (T/C)					
G: *UCP2* L: 11q13.4	Insertion/ deletion (I/D)	Malaysia (53) Case-control *N* = 926 Obese *n* = 265 Non-obese *n* = 661	PCR-RFLP ≥ 25 kg/m^2^	33.96 ± 8.51 Malay = 102 Chinese = 672 Indian = 152	*UCP2* 45-bp I/D polymorphism is associated with overall adiposity in Malaysian women	ID genotype associated with overall adiposity OR = 2.02 CI = 1.18, 3.45 *P* = 0.01
G: *PPARα* L162V L: 22q13.31	NR	Malaysia (54) Cross-sectional *N* = 307 Obese *n* = 18 Non-obese *n* = 127	PCR-RFLP ≥ 25 kg/m^2^	53.3 ± 14.2 Malay = 97 Chinese = 85 Indian = 55	No significant association of SNPs with obesity and metabolic syndrome *PPARα*, V162 allele carriers associate with high plasma IL-6 level	*PPARα*, L162V association with obesity and metabolic syndrome OR = 1.732 CI = 1.34, 8.77 *P* = 0.50
G: *PPARγ2* C161T L: 6p21.31	NR					
G: *PPARδ* T294C L: 3p25.2	NR					
G: *FTO* L:16q12.2	rs9939609	Indonesia (55) Case-control *N* = 80 Obese *n* = 38 Non-obese *n* = 40	ARMS PCR ≥ 25 kg/m^2^	32 Indonesian	Participants with the AT/AA genotypes reported to have 3.72 greater risk of obesity and 5.98 times higher risk for dietary fat intake	AT/AA genotypes associated with obesity OR = 3.72 CI = 1.19, 11.64 *P* = 0.009
G: *FTO* L:16q12.2	rs1421085	Indonesia (56) Case-control *N* = 71 Obese *n* = 35 Non-obese *n* = 36	ARMS PCR ≥ 25 kg/m^2^	32 (27.5 ± 36.8) Indonesian	CC genotype reported to show higher BMI TC/CC genotypes had higher monounsaturated and saturated fatty acid intakes than TT genotype	CC genotype has high BMI in recessive model Coef = 12.38 CI = 5.3, 19.46 *P* ≤ 0.001
G: *UCP2* L: 11q13.4	rs659366	Indonesia, Yogyakarta (57) Prospective cohort study *N* = 323	PCR-RFLP NR	42·8 ± 9·7 Indonesian	GG genotype showed more weight gain and influenced energy intake *UCP2* does not associated with adiposity in 2 2-year follow-up	GG genotype positively correlated with body weight *β* = 0·232 *P* = 0·016
G: *GHRL* L: 3p25.3	Leu72Met	Indonesia (58) Case-control study *N* = 198 Obese *n* = 100 Non-obese *n* = 98	PCR-RFLP ≥ 25 kg/m^2^	22.06 ± 4.08 Javanese	CA mutant genotype was found in 42.2% of obese group Leu72Met polymorphism increases the risk of obesity	Genotype AA+CA in obese OR = 4.96 CI = 2.43, 10.12 *P* ≤ 0.001
G: *FTO* L:16q12.2	rs9939609	Indonesia (59) Cross-sectional study *N* = 612 Normal *n* = 270 Overweight *n* = 218 Obese *n* = 121	ARMS PCR ≥ 25 kg/m^2^	46.6 ± 14.6 Balinese	The minor AA of rs9939609 and CC of rs1421085 increased BMI and were associated with obesity	rs1421085 is associated with high BMI OR = 3.22 CI = NR *P* ≤ 0.001
	rs1421085					
G: *LEPR* L: 1p31.3	rs1137100	Indonesia (60) Cross-sectional study *N* = 110 Obese *n* = 55 Non-obese *n* = 55	PCR-RFLP NR	20.7 ± 3.7 Indonesian	rs1137100 (K109R) and rs1137101 (Q223R) correlated with obesity and leptin level	LEPR, rs1137100 (K109R) genotype association with body weight OR = NR *P* = 0.000
	rs1137101					
G: *RETN* L: 19p13.2	rs3745368	Indonesia (61) Case-control *N* = 122 Obese *n* = 61 Non-obese *n* = 61	PCR-RFLP ≥ 27 kg/m^2^	22 Javanese	No association found between genotypes and alleles and obesity parameters and resistin levels	*RETN*, GG phenotype association with obesity OR = 2.07 CI = 0.36, 11.74 *P* = 0.680
G: *IL-6* L: 7p15.3	-174 G>C	Indonesia-western ethnic (62) Case-control *N* = 178 Obese *n* = 89 Non-obese *n* = 89	PCR-RFLP ≥ 25 kg/m^2^	22.1 ± 4.1 Indonesian	CC genotype had higher plasma CRP and lower *IL-6* levels than the GC and GG genotypes in obese group Obese population has more CC genotype than control	CC genotype associated with obese and control subjects OR = 7.39 CI = 2.26, 25.71 *P* = 0.0005
G: *UCP2* L: 11q13.4	rs660339	Indonesia (63) Case-control *N* = 200 Obese *n* = 100 Non-obese *n* = 100	PCR-RFLP ≥ 25 kg/m^2^	22.35 ± 4.81 Javanese	I/D genotypes were associated with obesity after gender stratification	Frequency of 45 bp II genotype in obese and control groups OR = 2.33 CI = 0.58, 9.40 *P* = 0.22
G: *UCP2* G(−866)A L: 11q13.4	rs659366	Indonesia (64) Cross-sectional *N* = 603 Obese *n* = 217 Non-obese *n* = 386	PCR-RFLP ≥ 25 kg/m^2^	49 ± 13 Balinese	No association between both *UCP* polymorphism and obesity traits Rural people with A/A genotype of G(−866)A has high BMI	OR = NR
G: *UCP2* Ala55Val (C>T) L:11q13.4	rs660339					
G: *UCP1* L: 4q31.1	rs1800592	Vietnam, Hai Duong (65) Case-control *N* = 140	PCR-RFLP ≥ 25 kg/m^2^	55.6 ± 3.8, Postmenopausal women Vietnamese	*VDR* polymorphisms, Basim and ApaI associated with overweight and obesity *VDR* and *ESR1* interaction reported to have effect on adiposity	*VDR ApaI* association with overweight and obese population OR = 3.00 CI = 1.08, 8.36 *P* = 0.03
G: *UCP2* L:11q13.4	rs659366					
G: *ADRA2B* L: 2q11.2	rs4994					
G: *LEPR* L: 1p31.3	rs1137101					
G: *ESR1* L: 6q25.1 q25.2	rs2234693 rs9340799					
G: *VDR* L: 12q13.11	rs2228570 rs1544410 rs7975232 rs731236					

I/D = insertion/deletion; OR = odds ratio; CI = confidence interval; NR = not reported; PCR = polymerase chain reaction; RFLP = restriction fragment length polymorphism; SNP = single nucleotide polymorphism; WHR = waist-to-hip ratio; HDL-C = high-density lipoprotein cholesterol; DBP = diastolic blood pressure; TG = triglycerides; BMI = body mass index; ARMS = amplification refractory mutation system

**Table 3:** The Newcastle-Ottawa Scale for the quality assessment of the selected case-control studies

Author	Selection				Comparability	Exposure			Total score
	Is the case definition adequate?	Representativeness of the cases	Selection of controls	Definition of controls	Comparability of cases and controls on the basis of the design or analysis	Ascertainment of exposure	Same method of ascertainment for cases and controls	Non-response rate	
Apalasamy et al. (45)	✵		✵	✵	✵	✵	✵	✵	7
Wan Rohani et al. (51)	✵		✵	✵	✵	✵	✵	✵	7
Lee et al. (52)	✵		✵	✵	✵		✵		5
Say et al. (53)	✵		✵	✵	✵		✵	✵	6
Chia et al. (54)	✵		✵	✵	✵	✵	✵	✵	7
Al-Jawadi et al. (56)	✵	✵	✵	✵	✵		✵	✵	8
Muhammad et al. (57)	✵	✵	✵	✵	✵		✵	✵	7
Priliani et al. (59)	✵	✵	✵	✵	✵	✵	✵	✵	8
Pramudji et al. (62)	✵	✵	✵	✵	✵	✵	✵	✵	8
Surniyantoro et al. (63)	✵	✵	✵	✵	✵	✵	✵	✵	8
Oktavianthi et al. (64)	✵		✵	✵	✵	✵	✵	✵	7
Binh et al. (65)							✵	✵	2

**Table 4:** The Newcastle-Ottawa Scale for the quality assessment of the selected cross-sectional studies

Author	Selection				Comparability	Outcome		Total
	Representatives of the sample	Sample size	Non-respondents	Ascertainment of the exposure (risk factor)	Comparability of studies on the basis of the design or analysis	Assessment of the outcome	Statistical test	
Fan et al. (41)	✵	✵	✵	✵	✵	✵	✵	7
Mohanraj et al. (42)	✵	✵	✵	✵	✵	✵	✵	7
Campbell et al. (43)	✵	✵	✵	✵	✵	✵	✵	7
Apalasamy et al. (45)		✵	✵	✵	✵	✵	✵	6
Lek et al. (46)	✵	✵	✵	✵	✵	✵	✵	7
Apidi et al. (48)		✵	✵	✵	✵	✵	✵	6
Mitra et al. (49)	✵	✵	✵	✵	✵	✵	✵	7
Kok et al. (50)	✵		✵		✵	✵	✵	5
Daya et al. (55)	✵	✵	✵	✵	✵	✵	✵	7
Hastuti et al. (60)	✵	✵	✵	✵	✵	✵	✵	7
Utami et al. (61)	✵	✵	✵	✵	✵	✵	✵	7

**Table 5:** The Newcastle-Ottawa Scale for the quality assessment of the selected cohort study

Author	Selection				Comparability	Outcome			Total
	Representatives of the exposed cohort	Selection of the non-exposed cohort	Ascertainment of exposure	Demonstration that outcome of interest was not present at start of study	Comparability of the cohort on the basis of the design or analysis	Assessment of the outcome	Was follow-up long enough for outcomes to occur	Adequacy of follow-up of cohorts	
Martantiningtyas et al. (58)	✵	✵	✵	✵	✵	✵	✵	✵	8
